# Inhibitory Effect on *In Vitro Streptococcus oralis* Biofilm of a Soda-Lime Glass Containing Silver Nanoparticles Coating on Titanium Alloy

**DOI:** 10.1371/journal.pone.0042393

**Published:** 2012-08-03

**Authors:** Belén Cabal, Fabio Cafini, Leticia Esteban-Tejeda, Luís Alou, José F. Bartolomé, David Sevillano, Roberto López-Piriz, Ramón Torrecillas, José Serafín Moya

**Affiliations:** 1 Nanomaterials and Nanotechnology Research Center-Spanish National Research Council-Universidad de Oviedo-Principado de Asturias, Parque Tecnológico de Asturias, Llanera, Spain; 2 Department of Biomaterials and Bioinspired Materials, Institute of Materials Science of Madrid-Consejo Superior de Investigaciones Científicas, Cantoblanco, Madrid, Spain; 3 Microbiology Department, School of Medicine, Universidad Complutense, Madrid, Spain; University of California, Merced, United States of America

## Abstract

This paper reports the effect of soda-lime-glass-nAg coating on the viability of an *in vitro* biofilm of *Streptococcus oralis*. Three strains (ATCC 35037 and two clinical isolates from periodontitis patients) were grown on coated with glass, glass containing silver nanoparticles, and uncoated titanium alloy disks. Two different methods were used to quantify biofilm formation abilities: crystal violet staining and determination of viable counts. The influence of the surface morphology on the cell attachment was studied. The surface morphology was characterized by scanning electron microscopy (SEM) and using a profilometer. SEM was also used to study the formation and the development of biofilm on the coated and uncoated disks. At least a >99.7% inocula reduction of biofilm respect to titanium disks and also to glass coated disks was observed in the glass-nAg coated disks for all the studied strains. A quantitative evaluation of the release of silver was conducted *in vitro* to test whether and to what extend the biocidal agent (silver) could leach from the coating. These findings suggest that the biofilm formation of *S. oralis* strains is highly inhibited by the glass-nAg and may be useful for materials which require durable antibacterial effect on their surfaces, as it is the case of dental implants.

## Introduction

The human oral cavity is a complex ecological environment where microorganisms have access to both hard and soft tissue surfaces to adhere, hence to develop biofilms. Dental plaque is a complex biofilm that accumulates on the tooth surfaces in the oral cavity embedded in a matrix of polymers [1]. The development and maturation of dental plaque as a biofilm has profound implications in the etiology and progression of the most prevalent infections affecting humans, namely, dental caries and periodontal diseases [2]. *Streptococcus oralis* is one of the most commonly detected early colonizers of the tooth surface as it has been demonstrated *in vitro* and *in vivo* studies [3], [4]. Primary colonizers alter the surface not only by their physical presence but also they are likely to represent a new “surface-attached” phenotype with distinct metabolic activity and surface properties, thus altering their surroundings and creating new niches for other bacteria to colonize [5].

Titanium dental implants are widely used because of its excellent biocompatibility and outstanding mechanical properties [6], [7]. Exposure of the implant in the oral cavity presents a unique surface that can interact with native host bacteria, leading to plaque formation. The development of materials with the ability to inhibit bacterial growth has been of great interest in recent years [8]. Inorganic antibacterial materials have several advantages over traditionally used organic agents; such as chemical stability, thermal resistance, safety to the user, long lasting action period, etc. [9]. Inorganic antibacterial materials are generally based on metallic ions with antibacterial properties, such as silver or copper, which are loaded into a ceramic matrix. In order to reduce the total silver or copper dose and thus to improve their biocompatibility, several investigations are currently developing nanostructured materials containing silver or copper nanoparticles [10], [11]. Nanostructured materials show a higher efficiency due to the large silver specific surface, which is inversely proportional to the particle size. This fact facilitates their applications in bioscience, medicine and other fields [12]. In a previous work [13], we have studied the feasibility to use soda-lime glass combined with uniformly distributed silver nanoparticles as novel antimicrobial coatings on titanium implants. Mechanically stable soda-lime coating (25 µm thickness) containing silver nanoparticles, ranging from 2.6–20 wt.%, on Ti-6Al-4V substrates were obtained by a simple sedimentation process at 980°C in Ar atmosphere. The coating containing 20 wt.% of silver nanoparticles has an excellent biocidal activity against Gram positive and negative bacteria and also against yeast. Based on those previous results, the present work is focused on assess the *in vitro S. oralis* adhesion to glass-nAg coating (20 wt.% nAg) of titanium alloy disks. Moreover, the controlled release effects of silver nanoparticles were evaluated. The release rate of a specific antimicrobial agent could has two opposite effects: for biocidal action, a fast release is preferred because it will lead to powerful and instant efficacies. On the other hand, however, the environmental impacts posed by releasing antimicrobial agents and the short-term effectiveness due to the exhaustion of these agents are also very important factors that should be taken into account in real applications.

## Materials and Methods

### Coatings of Titanium Alloy Disks

A thoroughly description of the procedure followed to coat titanium alloy plates and also the characterization can be found in our previous work [13]. Briefly, in a first stage homogeneous dispersed silver nanoparticles embedded into glassy matrix have been obtained as described below: A commercial soda-lime glass with the following chemical composition (wt.%): 70.20 SiO_2_; 15.80 Na_2_O; 7.10 CaO; 3.20 MgO; 1.71 Al_2_O_3_, 1.06 B_2_O_3_, 0.05 K_2_O and 0.02 Fe_2_O_3_, is homogenously mixed with the corresponding quantity of vitellinate/nAg (ARGENOL S.L.) to obtain a soda-lime-glass-nAg with 20 wt.% content of silver, then it is sintered in zirconia crucibles, in two-steps by heating at a rate of 3°C/min to 500°C for 1 h and to 725°C, for 1 h.

Disks (16 mm diameter x 3 mm thickness) were saw cut from a rod of 16 mm of diameter and 100 mm of length purchased by Good fellow (Ti017940/8, 99.0% purity). Cladding of these Ti-6Al-4V disks was performed by deposition of these powders (0.2 g), with a particle size distribution of d_50_ = 11.56±0.03 µm, from homogeneous suspension in acetone (20 mL) and subsequently air-dried at 40°C. Afterwards, the coated plates were heated in an argon atmosphere at 980°C for 1 h. Mechanically stable soda-lime coating (∼25 µm thickness) with (20 wt.% nAg) or without silver nanoparticles on Ti-6Al-4V substrates were obtained by a simple sedimentation process.

### Characterization

The silver-particle size was studied using transmission electron microscopy (TEM) (Jeol microscope model FXII operating at 200 kV.). Samples for TEM analysis were prepared by detaching pieces from the glass-nAg coated Ti-6Al-4V plate by bending and then milled in an agatha mortar down to 1 µm. The morphology of the coated or uncoated titanium alloy disks was studied before the biofilm tests by scanning electron microscopy (SEM) (Hitachi S-4300). After biofilm formation (see below), supernatants were removed, the disks were carefully washed with sterile saline solution to remove unattached cells, they were air-dried, and then characterized by SEM.

#### Surface roughness

In order to estimate the surface morphology, the sample surfaces were measured with a surface profilometer (Talysurf CLI 500, Taylor Hobson, Leicester, UK) that maps the surface by putting a stylus in mechanical contact with the sample. The stylus arm has 90° conisphere diamond styli with 2 µm nominal radius tip. The data sampling interval in X and Y was 0.5 µm and 2.5 µm respectively. The resolution (Z) was 32 nm. The profilometer was used to determine the three-dimensional topographic map and to calculate the roughness factor or specific surface area (the ratio of the surface to the projected area). Three samples of each surface type (coated and not coated) were scanned to evaluate the average surface roughness (R_a_) of the surfaces at five different locations.

### Bacterial Strains and Culture Conditions

Standard reference strain of *Streptococcus oralis* ATCC 35037 and two clinical isolates (CI-1 and CI-2) from a previous periodontal pathogen collection [14] were used in this study. Todd Hewitt Broth medium supplemented with 5% yeast extract (Difco; BD Diagnostics, Sparks, MD, USA) and 50 mM of glucose (Panreac, Barcelona, Spain) (THY-glucose) was the medium used in the biofilm experiements since in a preliminary study higher biofilms (data not shown) in THY-glucose than THY-saliva medium were observed in all strains.

### Biofilm Formation Assays

Colonies of *S. oralis* strains from an overnight culture on Columbia sheep blood agar (Difco, BD Diagnostic Systems, Sparks, MD, USA) were allowed to grow in THY-glucose at 37°C in the presence of 5% CO_2_ to a density of 0.5–1×10^8^ colony forming units (CFU/mL) as measured by a UV-Visible spectrophotometer (GBC, Model Cintra 101, Australia). Titanium disks (coated and not coated) were placed in 24-well culture plates and 100 µl of this inoculum suspension were inoculated in 900 µl (1∶10 dilution) of THY-glucose. Plates were incubated at 37°C in the presence of 5% CO_2_ for 24 h in a wet chamber.

Two methods were used to quantify biofilm formation abilities: i) crystal violet staining followed by measures of absorbance, and ii) determination of viable counts.

-The crystal violet assay was performed to determine the total amount of biofilm. After incubation, the disks were carefully washed three times with sterile saline solution to remove unattached cells. For fixation of biofilms, 300 µl of methanol was added. After 20 minutes, supernatants were removed and the disks were air-dried. Then, the biofilms were stained using 300 µl of 1% crystal violet solution (Química Clínica Aplicada, Tarragona, Spain), followed by 20 minutes incubation time at room temperature. Then, the excess of unbound dye was removed by washing the plates with water. The bound crystal violet was released by adding 200 µl of ethanol. The amount of biofilm was measured at optical density of 570 nm using a spectrophotometer. The background staining was corrected by subtracting the mean value for crystal violet bound to negative controls. All experiments were performed in triplicate.

-Viable counts were used to determine the quantity of viable adherent and planktonic (suspended) bacteria. After biofilm formation, supernatant samples of 200 µl were taken to determine viable planktonic bacteria and expressed as CFU/mL. Then, disks were carefully washed three times with sterile saline solution to remove unattached cells and inserted in tubes containing 5 mL of sterile saline solution. The tubes were vigorously vortexed for 2 minutes to free the bacteria attached on the surface of each disk and sonificated twice for 10 seconds (Microson ultrasonic cell disruptor XL Misonix, Inc., Fanningdale, NY, USA) to disperse bacterial cells. Samples of 200 µl from sonicated biofilms were serially diluted in 0.9% saline solution and plated onto Columbia sheep blood agar to determine the total number of viable cells and expressed as CFU/mm^2^. All experiments were performed in triplicate. The limit of detection was 2.5 × 10^2^ CFU. Preliminary experiments indicated that sonication was not associated with a loss of viability of the cells. Reduction of inocula was calculated by subtracting the log_10_ colony counts in the glass-nAg disks from those in the titanium disks. The percentage was calculated as %IR = 100 − (100 × In)/It, where *%IR* is percentage of inoculum reduction, *In* is the bacterial count (CFU/mm^2^) in the glass-nAg disks, and *It* is the inoculum (CFU/mm^2^) in the titanium disks.

### Statistical Analysis

All statistical analyses were performed by ANOVA with the Tukey test for multiple comparisons. A P-value of <.01 was considered statistically significant.

### Determination of Released Silver

The release rate of silver from soda-lime glass-nAg coating of titanium alloy disks was measured as follows: A randomly selected set of five coated disks was studied during a period of 7 days. Each disk was incubated in 1 mL of THY-glucose using the same conditions of the biofilm formation experiments. At defined time intervals of 24 h, the entire broth content was taken and the released silver was measured by inductively coupled plasma (ICP) (ICP Perkin Elmer mod. Optima 2100 DV). After each measurement, the media was renewed. The cumulative concentration of silver was obtained and plotted versus time.

## Results

### Characterization

**Figure 1:**
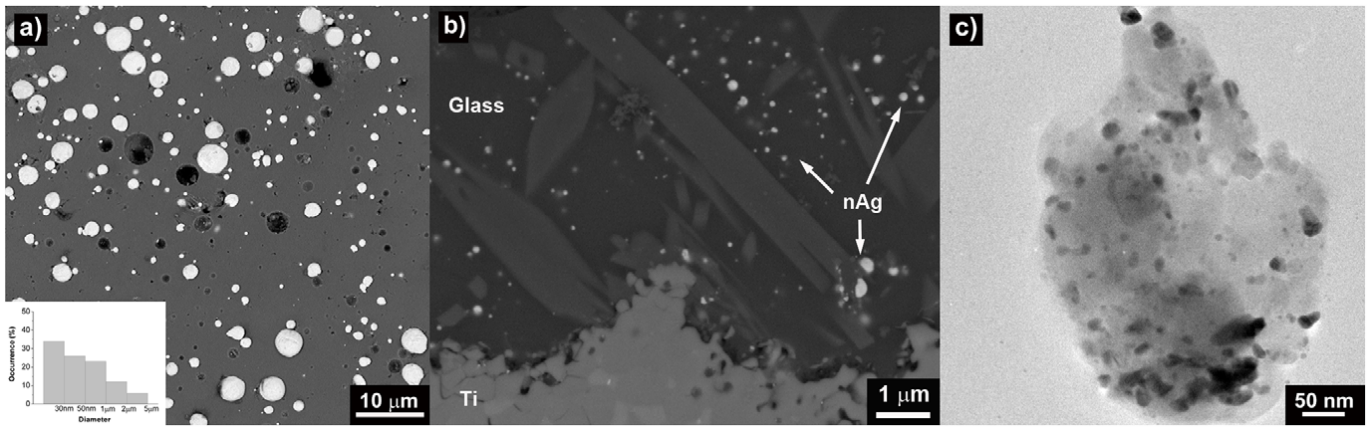
Scanning electron micrographs of titanium alloy disk coated with glass-nAg before the biofilm tests at different magnifications: a) top view, b) cross section. c ) TEM micrograph of glass-nAg coating. Inset shows the size distribution of silver nanoparticles obtained from SEM and TEM micrographs analysis.

The microstructure of the soda-lime-glass-nAg coating on Ti-4Al-6V disk is shown in figure 1(a and b). Silver nanoparticles are evenly embedded into the glassy matrix coating (figure 1.c). A size distribution of globular-shaped silver nanoparticles ranging between 20–90 nm but also some agglomerates (0.5–8 µm) are present. The process followed to coat the disks did not agglomerate significantly the silver nanoparticles.

#### Surface roughness

**Figure 2:**
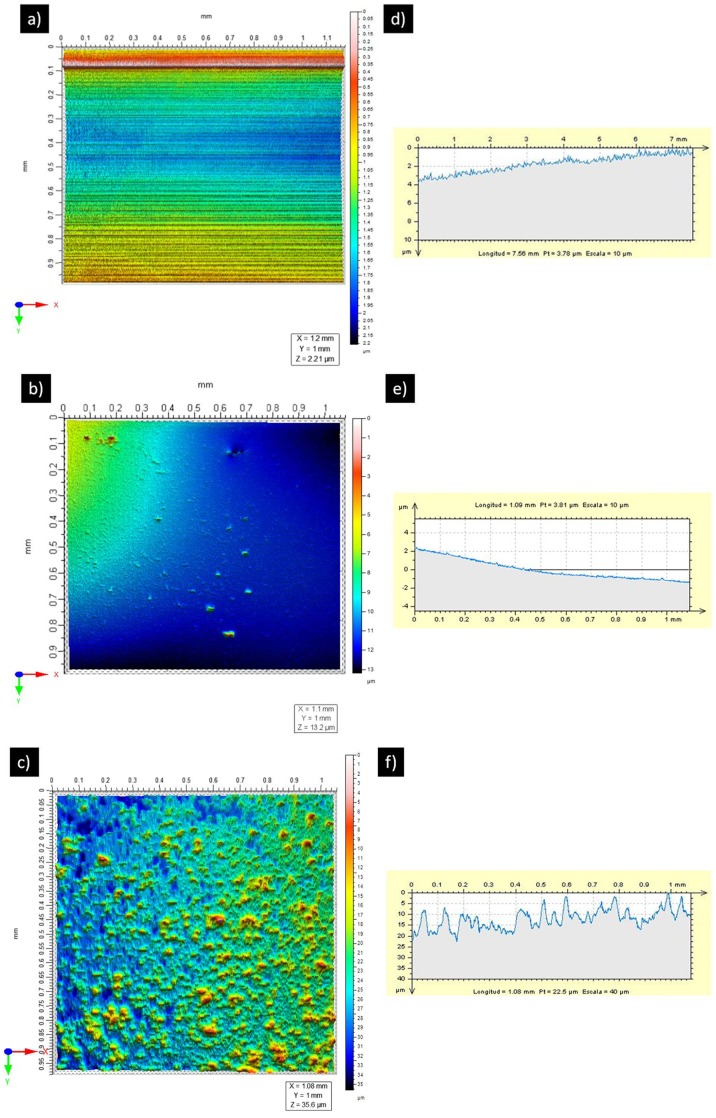
Profilometer characterization of surface topography of the different samples. Representative height maps in three-dimensional view of: a) uncoated titanium alloy disk, b) glass coated Ti4Al6V disk c) glass-nAg coated Ti4Al6V disk. Representative surface profiles exhibiting variations in R_a_ of: d) uncoated titanium alloy disk, e) glass coated Ti4Al6V disk and f) glass-nAg coated Ti4Al6V disk. The images correspond to 1 mm×1 mm scan area.

Representative 3D surface topographies of the samples with corresponding surface profiles are shown in figure 2. The figures clearly show that the glass-nAg coated materials present a micrometrical roughness, whereas the Ti-4Al-6V surfaces (glass coated or not) presented a R_a_ in nanometrical scales. Significantly higher average R_a_ values were found for glass-nAg coated disks (3.12±0.43 µm) than for titanium disks (0.08±0.03 µm) and also for glass coated disks (0.11±0.04 µm). The same trend was observed in the values of specific surface area (Aspec). It is always larger than unity, however, in the case of glass-nAg coated disks, this value is near two times higher (1.8±0.2) than for titanium disks (1.02±0.01) and for glass coated disks (1.08±0.02). The obtained data evidenced a higher roughness and specific surface area on glass-nAg coated surfaces compared with titanium alloy disks.

### Biofilm Formation

**Figure 3:**
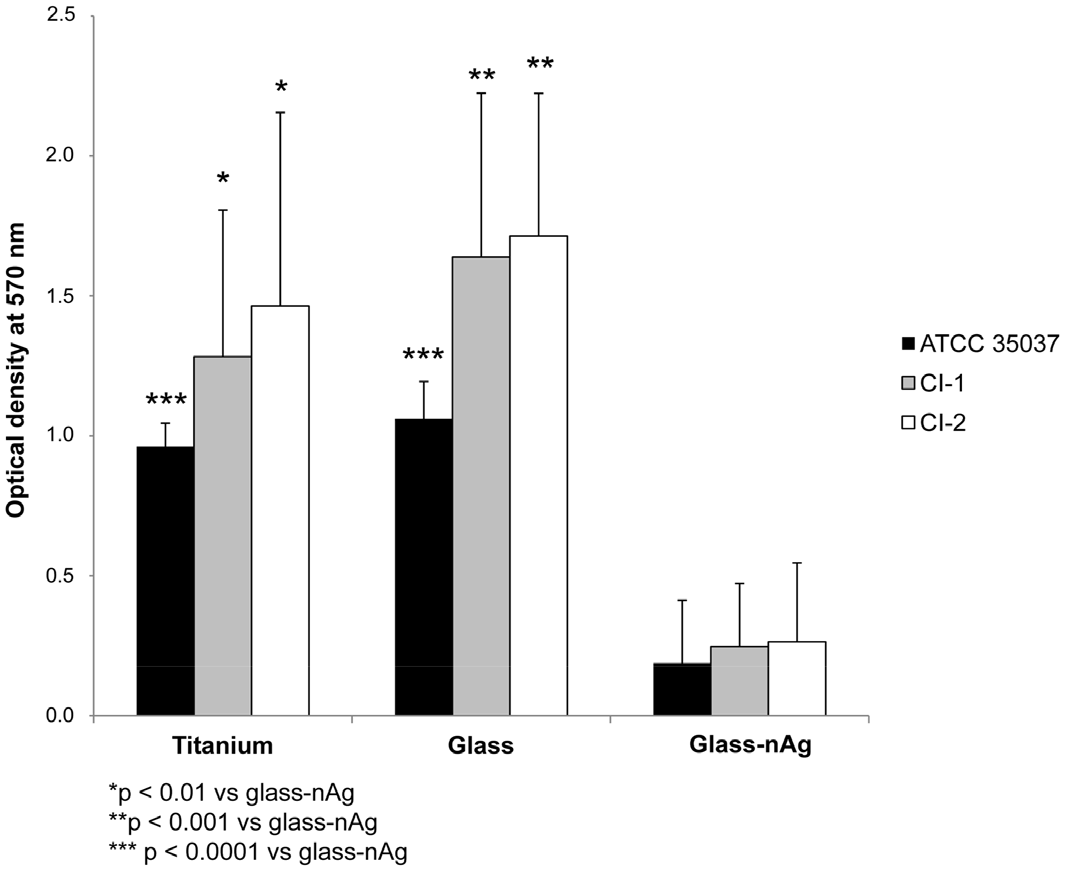
Comparison of total biofilm mass for the studied S. oralis strains determined by crystal violet staining among coated (glass or glass-nAg) and uncoated titanium alloy disks.

**Figure 4:**
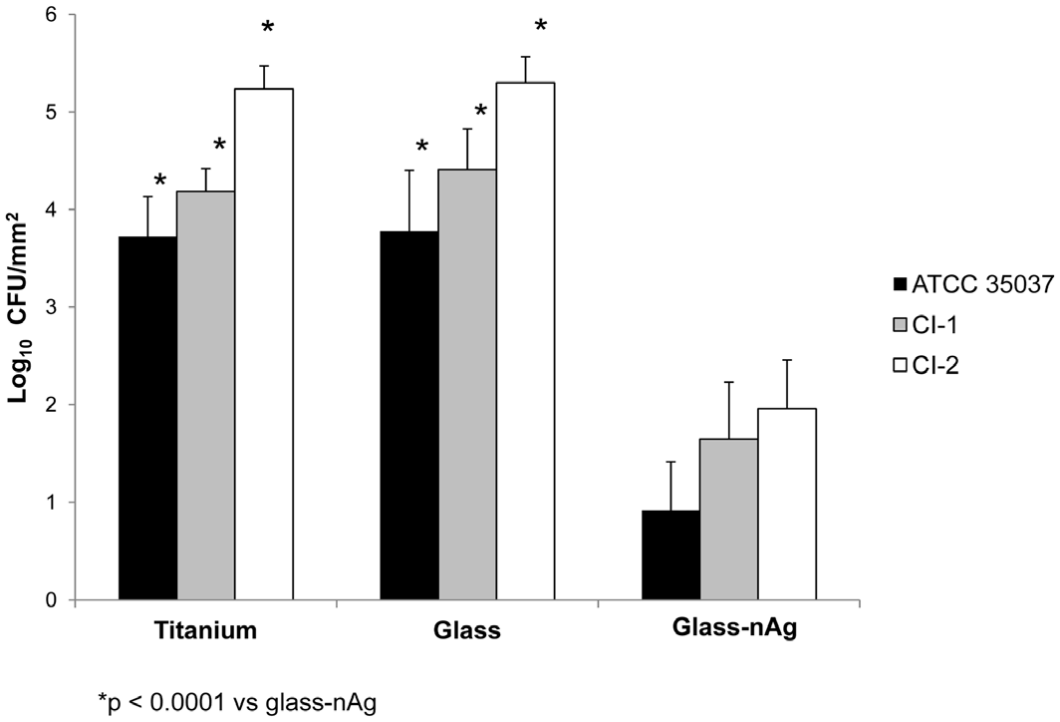
Comparison of the viable adherent bacteria (CFU/mm^2^) for the studied S. oralis strains among coated (glass or glass-nAg) and uncoated titanium alloy disks.

Figure 3 shows the total biofilm mass for all *S. oralis* strains determined by crystal violet staining. Absorbance values from titanium and from glass coated disks were significantly higher (p<0.01) than those from glass-nAg disks, for all strains. No significant differences were observed between titanium and glass disks. Figure 4 shows the adherence of the viable *S. oralis* strains (log_10_ CFU/mm^2^) to titanium, glass and glass-nAg surfaces. Colony counts from titanium and glass disks were significantly higher (p<0.0001) than those from glass-nAg disks for all *S. oralis* strains. The coating with glass-nAg significantly reduced the adherence of *S. oralis* ATCC 35037, CI-1, and CI-2 with respect to titanium and glass surfaces by at least 99.8, 99.7, and 99.9% at 24 h, respectively. No significant differences were observed between titanium and glass coated disks.

**Figure 5:**
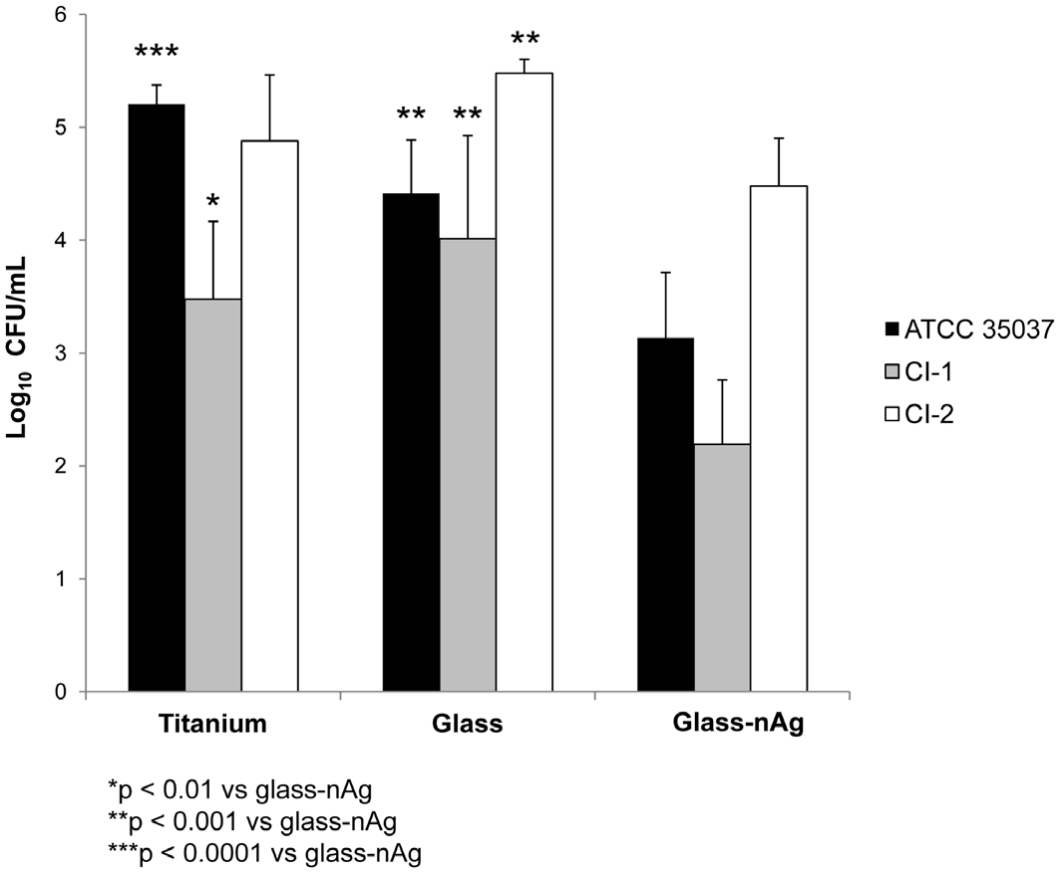
Comparison of the viable planktonic bacteria (CFU/mL) for the studied S. oralis strains among coated (glass or glass-nAg) and uncoated titanium alloy disks.

Figure 5 shows the viable planktonic cells (suspended) of the *S. oralis* strains (log_10_ CFU/mL) in THY-glucose after 24 h incubation in the titanium, glass, and glass-nAg surfaces. Colony counts of planktonic (suspended) cells from the titanium and glass coated disks were significantly higher than those from the glass-nAg coated disks in all *S. oralis* strains except for the titanium *versus* glass-nAg in the CI-2 strain. No statistically significant differences were observed in the colony counts of planktonic cells for the CI-1 and CI-2 strains. No significant differences were observed between titanium and glass coated disks.

**Figure 6:**
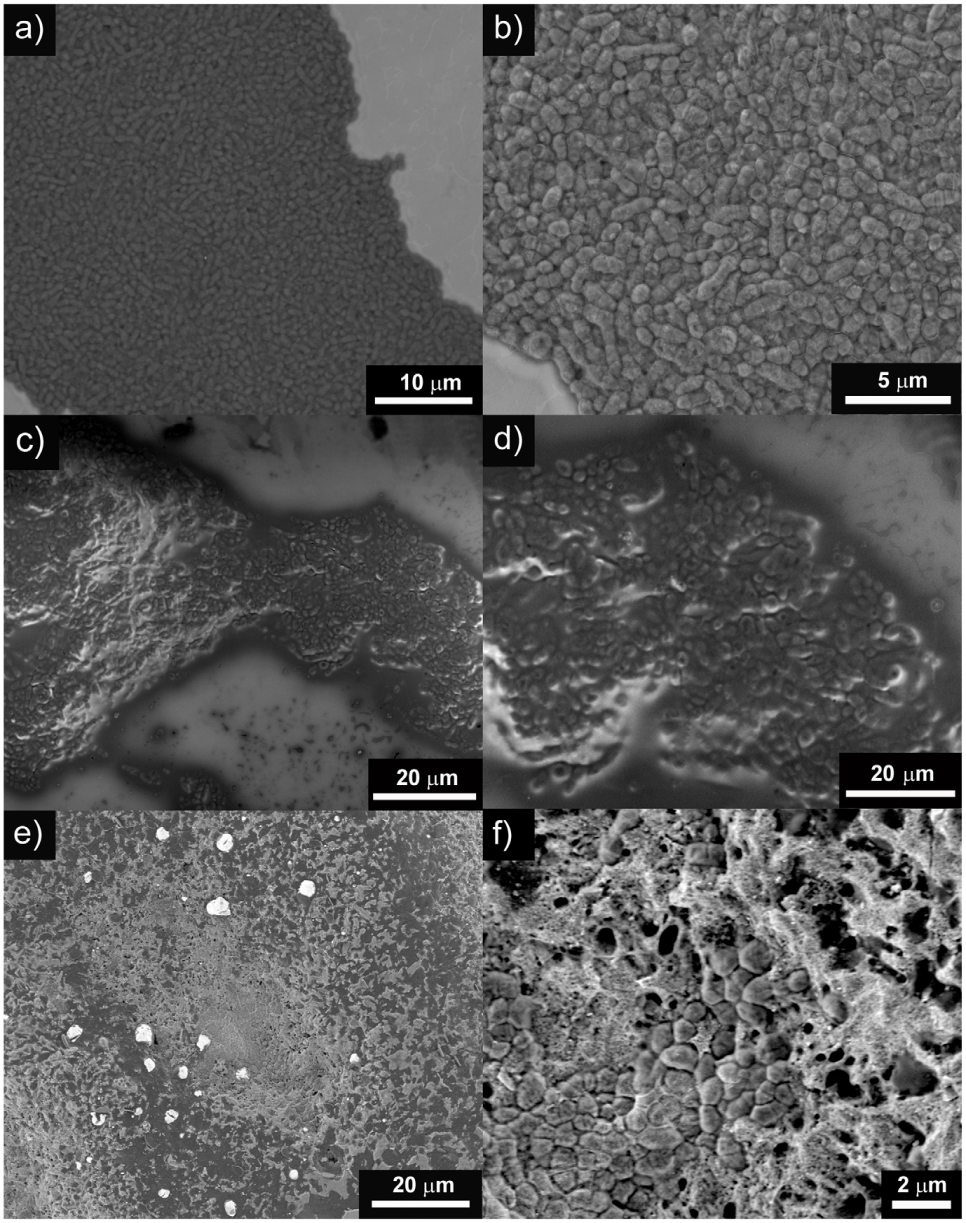
Scanning electron micrographs at different magnifications after biofilm formation of the CI-1 strain on: a, b) uncoated titanium alloy disk; c, d) titanium alloy disk coated with glass; and e, f) titanium alloy disk coated with glass-nAg.

Scanning electron microscopy was also used to study the formation and the development of biofilm on the coated and uncoated disks (Fig. 6).

### Silver Release

**Figure 7:**
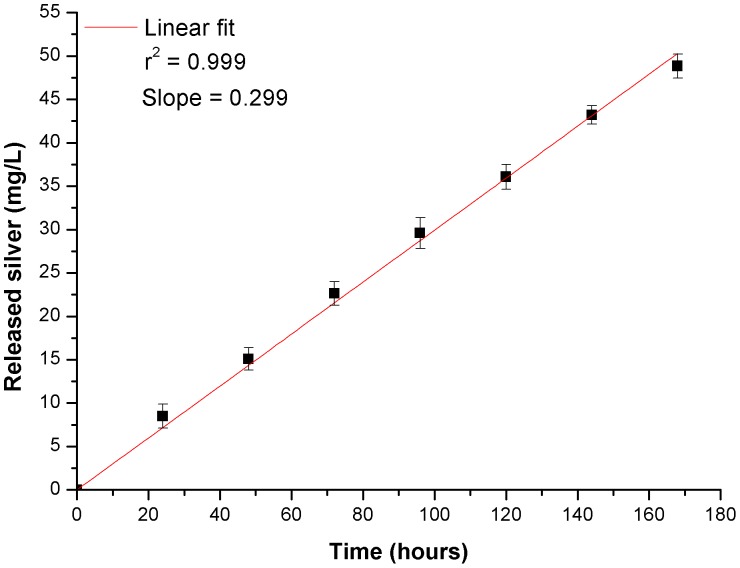
Average cumulative silver released from glass-nAg coating of titanium alloy disks in the culture at 37°C as a function of time.

The amount of silver released into supernatant from the glass-nAg coated disks was determined for different time intervals up to 7 days. The average of the obtained results is shown in figure 7. As it is clearly seen, the amount of released silver is proportional to the soaking period. The release rate was 0.299 mg L^−1^ h^−1^, calculated by fitting a straight line through the data.

## Discussion

The physico-chemical characteristics of specific material surfaces are known to significantly influence the bacterial adhesion process [15]. The surface roughness has been suggested to play a major role in this process [16], [17]. Roughened surfaces promote bacterial adhesion and biofilm formation more than ultrasmooth surfaces. This may happen since a rough surface has a greater surface area and the depressions in the irregularities provide more favorable sites for colonization. In our study, despite higher R_a_ value (i.e., 3.12 µm) and higher specific surface area (ca., two times higher), of the glass-nAg surfaces with respect to that of titanium surface (i.e., 0.08 µm), and also versus silver glass free surfaces (i.e., 0.11 µm), significantly lower microbial quantity of adhering *S. oralis* on the glass-nAg coated disks was found by both methods used (crystal violet staining and colony counts). This result demonstrated that the biofilm formation is highly inhibited by the glass-nAg coating.

The ability of glass-nAg coating to reduce viable bacteria colonization and to prevent biofilm formation has been also verified by scanning electron microscopy as shown in Fig. 6. Representative SEM images of the uncoated and coated (glass and glass-nAg) titanium alloy disks surfaces after 24 h incubation with CI-1 are shown. It can be seen that the bacterial cells colonized densely onto the surface of the uncoated titanium alloy disk (Fig. 6 a and b) and onto glass coated disks (Fig. 6 c and d). Conversely, the extend of bacterial adhesion on the glass-nAg coating decreased significantly and only a few microorganisms appear located inside the porous of the coating (Fig. 6 f and d). This fact clearly state that glass-nAg reduces bacterial adhesion, therefore, avoids the biofilm formation, and it is consistent with the quantification of biofilm formation. Similar results were obtained with ATCC and CI-2 strains (data not shown).

The biocide action of silver nanoparticles is also reflected in the planktonic (not adhered) cells. A diminishment of colonies was recorded for all the studied strains when the glass-nAg coated disks were used, being more significance in the case of the reference strain (Fig. 5).

On the other hand, particular attention must be paid to the toxicity issues surrounding the potential impact of silver nanoparticles on oral and other tissues. The concentration of released silver in the supernatant at 24 h was ca. 7 ppm, that is below the cytotoxic level in human fibroblast previously described (i.e., 30 ppm) [18]. The silver release profile (Fig. 7) gives an indication of how well controlled the release of silver from the coating is. Taking into account that the silver nanoparticles are homogeneously distributed in the glassy matrix (Fig. 1), its release rate is directly dependent on the overall rate of the glass dissolution. The glass dissolution usually proceeds by two main types of chemicals reactions depending on whether the cation occupies a network forming or modifying site. During the first stages, known as leaching or selective dissolution, network modifiers (Na^+^, K^+^, Ca^2+^) are selectively extracted from the glass surface. This type is addressed by a parabolic relation between the weight lose and the time. In the next stages, known as etching or network dissolution, network forming cations (Si^4+^, B^3+^, Al^3+^) pass into the aqueous solution as a result of the breakdown of the glass network structure at the leached layer solution interface. This type is addressed by a linear relation between the weight loss and time [19].

In the case of this soda-lime glass used as matrix of silver nanoparticles, the hydrolytic resistance of this soda-lime glass (i.e., ≤2 [g · (cm^2^ · s)^−1^×10^−8^]) was studied in previous works. The weight loss during its dissolution in water was found to be proportional to the time of glass dissolution, having a constant dissolution release rate [20], [21]. This behavior of glass dissolution clearly indicates that the mechanism of total dissolution of the glass network with no selective leaching of cations from glass is the predominating mechanism of dissolution. Consequently, we can stated that the soda-lime-glass-nAg used in the present investigation is a very promising coating for materials which require durable antibacterial effect on their surfaces, as it is the case of dental implants. The main advantages that this glass-nAg offers in comparison with other biocides reported in the literature are that not only enables a controlled sustained delivery of the antimicrobial agent (silver nanoparticles) as well it provides long-term action.

### Conclusions

In this study, soda-lime-glass-nAg coating was found to effectively inhibit the formation of *in vitro Streptococcus oralis* biofilm. SEM studies confirmed the results of the quantitative biofilm colonization models and fully desmostrated the efficacy of this coating. The novelty of the work is that using this soda-lime glass coating as a matrix to dosify silver nanoparticles, the inherent agglomeration of the metal nanoparticles is avoided and at the same time, it enables a controlled sustained delivery of them as well as it provides long-term action. The quantitative evaluation of silver release pointed out the low toxicity and long-term effectiveness of the coating. This method provides a promising strategy with respect to the fabrication of durable antibacterial surfaces.
